# A scanning electron microscopy study of *Anisakis physeteris* molecularly identified: from third stage larvae from fish to fourth stage larvae obtained in vitro

**DOI:** 10.1007/s00436-018-5896-5

**Published:** 2018-05-07

**Authors:** Dolores Molina-Fernández, Francisco Javier Adroher, Rocío Benítez

**Affiliations:** Departamento de Parasitología, Facultad de Farmacia, Universidad de Granada, 18071 Granada, Spain

**Keywords:** *Anisakis physeteris*, Third larval stage, Fourth larval stage, Scanning electron microscopy, Morphology, Development, Anisakiasis

## Abstract

The development of the fourth larval stage (L4) of *Anisakis physeteris* was studied using scanning electron microscopy (SEM), comparing it with third larval stage (L3) recently obtained from the host fish, blue whiting (*Micromesistius poutassou*), from the western Mediterranean Sea (east coast of Spain, zone FAO 37.1.1). After molting to L4, samples of the parasite were examined at different times in order to observe their development. Following collection of the L4, a small portion was taken from the middle of the larva for molecular identification, confirming in all cases that it was *A. physeteris*. The anterior and posterior sections of the larvae were prepared for morphological study by SEM. The development of a row of denticles on each of the three prominent lips, almost reaching the buccal commisures, was observed in the L4. Pores of unknown function were found in the upper external part of each lip. Clearly developed cephalic papillae, amphids, and deirids were also observed in L4, while, although present in L3, these were beneath the cuticle. Phasmids were detected in L4 but not in L3. The L4 tail finished in a conical lobe with a blunt point, absent in L3. In the oldest L4, some preanal papillae were observed beneath the cuticle in males, while, in females, the vulva could be seen by light microscopy, apparently still covered by the cuticle.

## Introduction

Nematodes of the genus *Anisakis* are parasites of the digestive tract of cetaceans. They are present in all the oceans and have a complex life cycle which also includes crustaceans, squid, and fish. The eggs, which are released into the sea in the feces of the definitive host, develop by hatching into the third larval stage (L3), which infects marine invertebrates, particularly euphausiids, the first intermediate hosts of *Anisakis* spp. These crustaceans are then ingested by squid and/or fish, which act as intermediate/paratenic hosts. The L3 then grow until they are infective for the definitive host, but without changing stage. The ingestion of these infected fish or squid by suitable cetaceans allows the L3 to develop into adults in their digestive tract. In the case of *Anisakis physeteris*, squid seem to have an epidemiological role as intermediate/paratenic hosts as they are an important source of food for sperm whales (95% or more of the diet; Santos et al. 2001 and references therein), the definitive host of this species (Baylis 1923; Kagei et al. 1967; Mattiucci and Nascetti 2008).

In L3 of the genus *Anisakis*, there are two morphological types of larva. Berland (1961) classified them as type I (elongated ventriculus with oblique join to the intestine, tail with mucron) and type II (shorter, thicker ventriculus with straight join to the intestine, conical tail), with the species within each type being morphologically indistinguishable. Molecular studies are gradually clarifying the taxonomy of these and other anisakids. Mattiucci et al. (2018) have divided the genus *Anisakis* into four clades. The species with type II L3 were included in clade 3 with three species identified to date: *A. physeteris*, *A. paggiae*, and *A. brevispiculata*.

Although anisakid L3 and their adult forms have been accurately described, the descriptions of their fourth larval stages are frequently lacking in detail. The aim of the present study is to fill in these gaps, using SEM to study the differences between L3 collected from the host fish and the L4 of *A. physeteris*, obtained in vitro, and their evolution during development. In spite of most cases of human anisakiasis being caused by larvae of the *A. simplex* s.l. complex (Rello Yubero et al. 2004), this study is of particular interest since *A. physeteris* is also able to cause it. Both L3 (Asato et al. 1991) and L4 (Clavel et al. 1993) have been collected from patients, in addition to L3 molting to L4 (Kagei et al. 1978).

The present study describes the third and fourth larval developmental stage of *A. physeteris* using scanning electron microscopy (SEM) and aims to improve understanding of the morphological characters of biological and taxonomical significance of this anisakid.

## Materials and methods

### Collection of parasites

L3 of *Anisakis* spp. were collected from blue whiting landed at the ports of Villajoyosa, Castellón and Gandía (western Mediterranean Sea, east coast of Spain, zone FAO 37.1.1). The fish were transported to the laboratory under refrigerated conditions and then dissected. The larvae, encapsulated in the visceral cavity, were collected and placed in a cold solution of NaCl 0.9%. They were then classified morphologically as type I or II sensu Berland (1961) using optical microscopy.

### Cultivation of parasites

Following morphological identification of the type II larvae of *Anisakis*, these were axenized in antibiotic-antimycotic solution (Iglesias et al. 1997) and individually placed in culture. The L3 measured between 2 and 3 cm in length. Both the culture medium and the procedure were described previously by Iglesias et al. (2001) for *A. simplex*. The parasites remained in culture until attaining the level of development required for the study, with any larvae failing to complete the molt to L4 being discarded. The larvae were examined daily by optical microscope to monitor their degree of development, mobility, and the sterility of the culture.

### Collection of larvae for SEM

After completing the molt to L4, the larvae were removed from the culture for the SEM study of 1–9 weeks. In addition, L3 recently collected from the fish were also examined by SEM for comparison with the L4. All larvae collected were fixed in hot 70% (*v*/*v*) ethanol and preserved for preparation for examination by SEM. The fixed larvae underwent critical point drying and cut into 3 sections, thus avoiding the distortion of the larvae which occurs if cut when fresh or only fixed. The anterior and posterior sections were separated for SEM preparation while a small cylindrical part of the central section was used for molecular identification.

### Molecular identification

The extraction of the genomic DNA from the central section of each larva was carried out using the RealPure (REAL) kit, according to the manufacturer’s instructions. Amplification of the region ITS1-5.8S-ITS2 of the ribosomal DNA was performed using the primers NC5 (forward) and NC2 (reverse) described by Zhu et al. (1998). The polymerase chain reaction procedure (PCR) was carried out as previously described in Molina-Fernández et al. (2015). The expected size of the amplicon was around 1000 bp. Next, a restriction fragment length polymorphism (RFLP) of the amplified DNA was carried out using the restriction enzymes *HinfI* (final concentration 0.5 U/μl, temperature 37 °C for 10 min) and *TaqI* (0.5 U/μl, 65 °C for 10 min) (Fast Digest, Thermo Scientific). To identify the species, electrophoresis with 3% agarose gel was performed to visualize of the band patterns of the larvae. The controls of the digestion by *TaqI* of the DNA amplicon of *A. physeteris* produced 3 fragments of 300, 280, and 140 bp. When digestion was with *HinfI*, the fragments were of 380, 290, and 270 bp, according to D’Amelio et al. (2000), Romero et al. (2014) and Molina-Fernández et al. (2018).

## Results and discussion

All larvae prepared for SEM (2 L3 and 8 L4) were analyzed molecularly and identified as *Anisakis physeteris* (Baylis, 1923). The L4 were obtained after 15–20 days of culture.

### Anterior or cephalic end

**Fig. 1:**
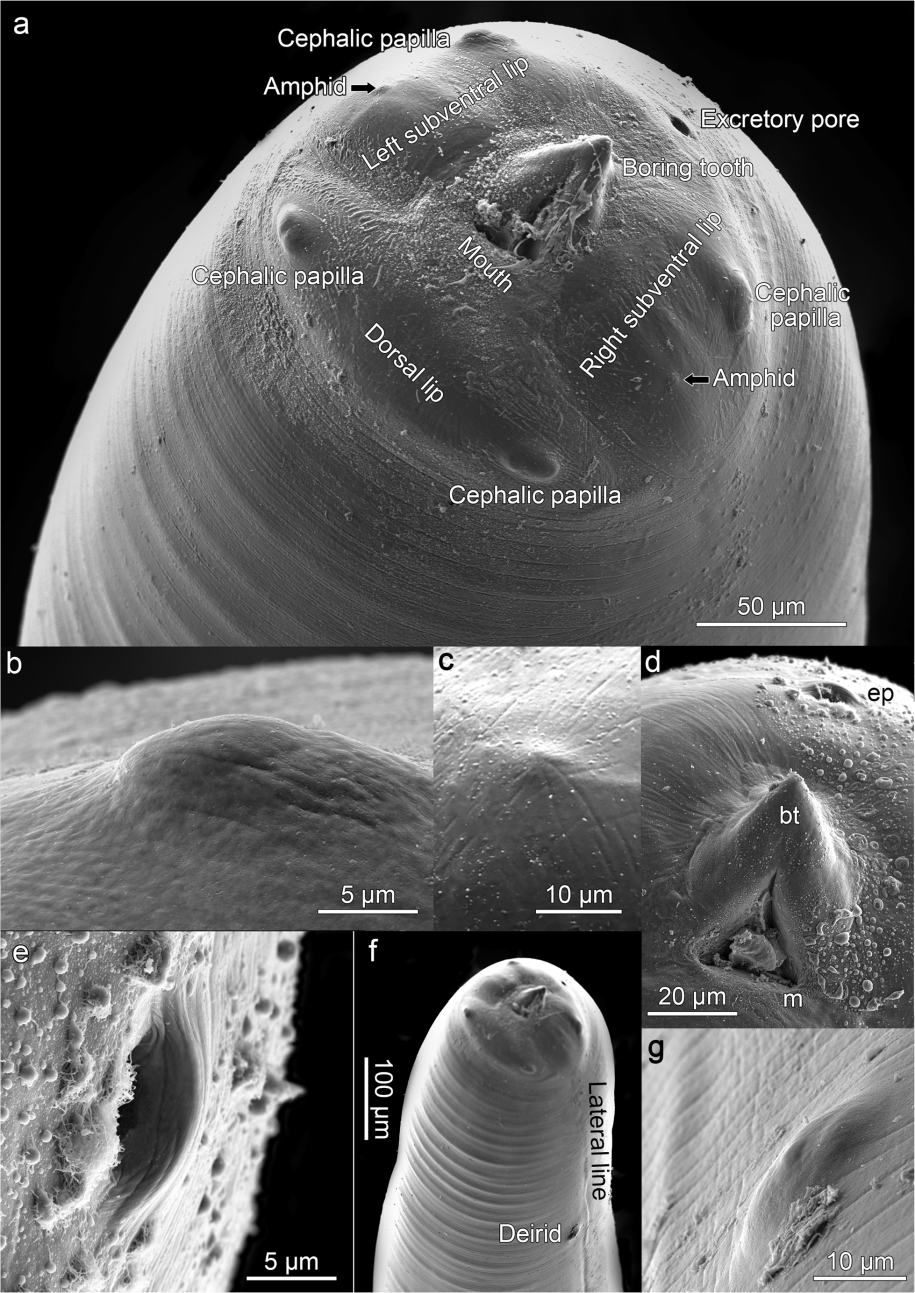
*Anisakis physeteris* L3. **a** Anterior end. **b–g** Detail of the structures: **b** Cephalic papilla. **c** Amphid. **d** Mouth (m), boring tooth (bt) and excretory pore (ep). **e** Excretory pore. **f** Location of deirid and lateral line in anterior end of larva. **g** Deirid and lateral line

**Fig. 2:**
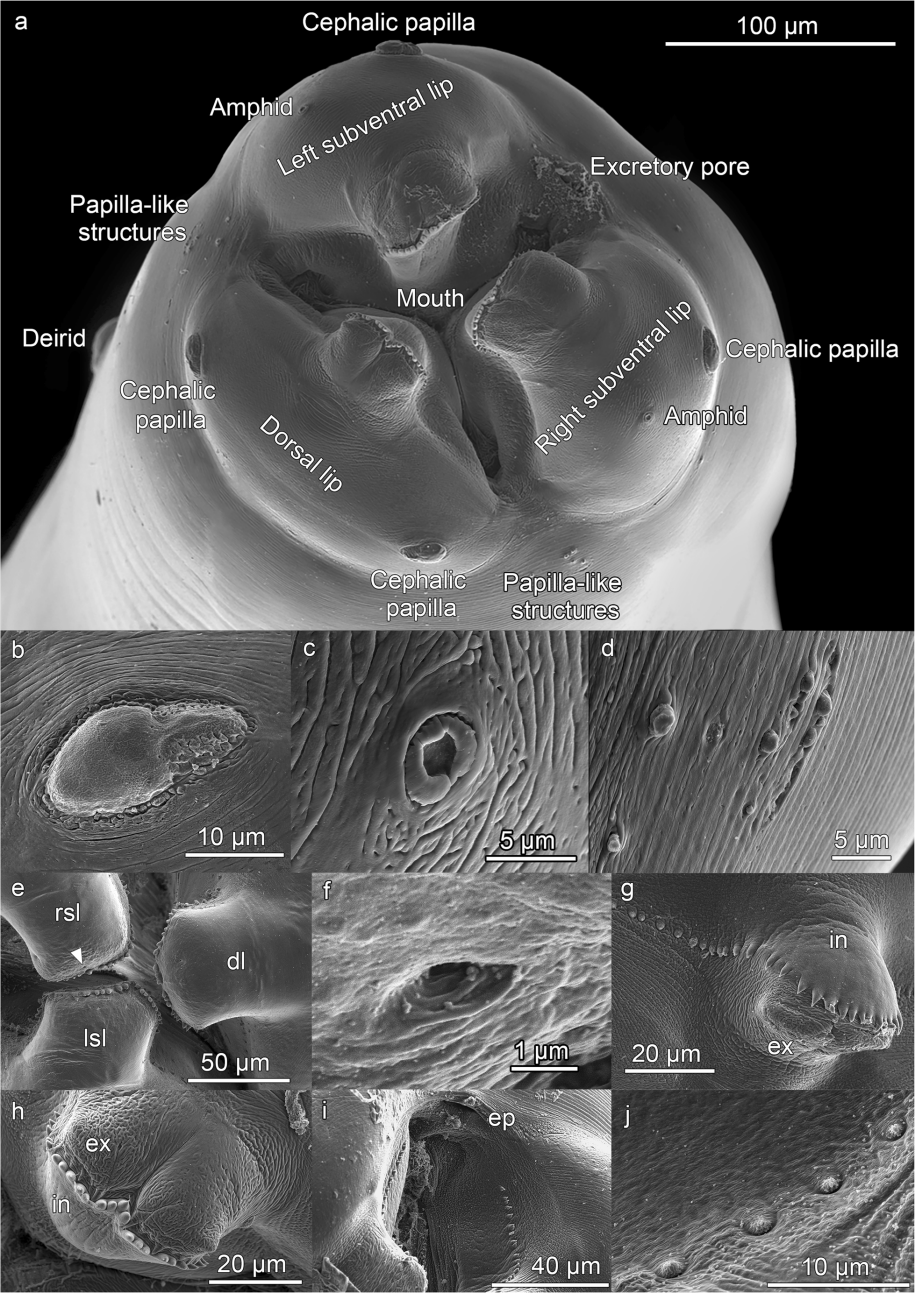
*Anisakis physeteris* L4. **a** Anterior end. Note the upper part of the lip, bilobed but symmetric in the dorsal lip and asymmetric in the subventral lips. **b–j** Detail of the structures of cephalic end: **b** Cephalic papilla. **c** Amphid. **d** Papilla-like structures located between the base of the dorsal and subventral lips (see in **a**). **e** Face view of the L4 with the three denticulate lips (dorsal (dl) and right (rsl) and left (lsl) subventral lips), observe the shape of the lips that allows them to fit; note pores in lips. **f** Pore of the right subventral lip marked with arrowhead in **e**, magnified. **g** Row of denticles, continuation of denticulate ridge, along side of upper part of lip and following the line of the mouth; (ex) external and (in) internal lip surfaces. **h** Upper part of dorsal lip (viewed from above), bilobed, with denticulate ridge, note rough external (ex) and smooth internal (in) surfaces. **i** Mouth commissure between subventral lips, note excretory pore (ep) opening towards mouth aperture and row of denticles reaching almost to the commisure. **j** Small hemispherical denticles located at end of rows

At the anterior end of anisakid L3 larvae, it is traditional to describe the presence of 3 lips or fairly pronounced labial protuberances with papillae or papilla-like structures, 2 on the dorsal lip and 1 on the 2 subventral lips. In *A. physeteris*, 3 incipient lips could be seen with 2 protuberances on each (Fig. 1a), as in *Anisakis* type I (Valter et al. 1982; Weerasooriya et al. 1986), covered by the cuticle and corresponding to the cephalic papillae and amphids (Fig. 1b, c) which are observed after molting to L4. The observation that these sense organs are covered by the cuticle in L3 poses the question as to whether they are functional or not and whether their development in L3 is only preparation for L4. However, the observation of undifferentiated papilla-like structures on the labial protuberances of recently hatched L3 of other anisakids (McClelland and Ronald 1974; Molina-Fernández et al. 2017) suggests that these structures may have some function in L3. Jones (1994) proposed that at least the amphids of L3 of *A. simplex* (s.l.) are functional since their internal structure is well-developed and this may also be true for the papillae. The mouth (Fig. 1a, d) is triradiate but opens to form a triangular aperture, as also occurs in L4 (Fig. 2). The boring tooth (Fig. 1a, d) is found surrounding these radia, between the two subventral lips. Below the boring tooth, in a ventral position, is an oval excretory pore with a maximum diameter of 7–10 mμ with its mouth pointing forwards (Fig. 1a, e) and appearing to empty towards the exterior of the larva. The deirids (also called body papillae, cervical papillae or lateral cervical papillae) are covered by the cuticle but visible in L3 (Fig. 1f, g), as are the papillae and amphids. These deirids are in a subdorsal position, close to the lateral lines running along the larva. Davey (1971) considered deirids to be present in specimens of the genus *Anisakis*, at least from the L3 stage, as found in the present study.

The L4 does not have a boring tooth but has 3 developed lips, the dorsal lip being clearly larger than the 2 subventral lips (Fig. 2a). A large, elongated cephalic papilla (Fig. 2a, b) is located laterally (towards the other subventral lip) at the base of each subventral lip. The structure of the papillae is similar to that described in the adults of other *Anisakis* spp. (Weerasooriya et al. 1986; Abollo and Pascual 2002; Di Azevedo et al. 2015). There is also an amphid (4.3–6.3 mμ diameter), with an annular structure, on each subventral lip, on the side close to the dorsal lip (Fig. 2a, c). The amphid is surrounded by irregular cuticle. The dorsal lip shows 2 elongated cephalic papillae, opposite each other at the base and has no amphids (Fig. 2a). These papillae (Fig. 2b) are of a similar size to those of the subventral lips, their length increasing with larval development time (from 18.2 to 32.8 mμ) and maintaining the width (10.4–12.7 mμ). They are surrounded by a more regular cuticle than that surrounding the amphid (Fig. 2b, c). At the anterior end, other papillae-like structures of unknown function, varying between individuals, can occasionally be observed (Fig. 2a, d). At the external upper part of the three lips, small pores of unknown function can be seen (Fig. 2e, f). This character does not appear to have been described previously in anisakids. As the L4 develops, the upper part of the lips can be seen to become more bilobed, clear in type I L4 of *Anisakis* (Weerasooriya et al. 1986) and in adults of the genus *Anisakis* (Davey 1971), shown by a cleft which is not always visible (Fig. 2a, e, h). The lobes are symmetrical in the dorsal lip but not in the subventral lips, allowing them to fit together (Fig. 2a, e). The lips are crowned by a ridge of denticles, as in type I *Anisakis* (Weerasooriya et al. 1986), which continues along the side of the lips with the denticles becoming progressively smaller and more rounded (possibly due to their being less developed) and almost reaches the commisures of the triradiate mouth (Fig. 2a, e–j). The number of denticles seems to vary between lips and individuals, roughly ranging from 40 to 65 per labium, the largest being those of the ridge. Weerasooriya et al. (1986), for *Anisakis* type I, reported between 35 and 40 only in the ridge on the lip and did not mention the presence of denticles outside the ridge. In the ridge of *A. physeteris*, between 13 and 19 simple denticles were counted, blunt or pointed, and, occasionally, double, while in type I *Anisakis*, the denticles were saw-toothed. In this sense, it has been observed that *A. pegreffii* only has denticles in the ridge, many forming a saw with no separation between them (personal observation). A total of 278 denticles (90–94 per lip) were counted in adults of *A. physeteris* (Kikuchi 1974), although the species identification was only morphological and it is not known whether this parameter varies between the 3 species of clade 3. The oval excretory pore is located between the subventral lips, as in L3, although in L4, it is within the area of influence of the mouth, thus allowing it to empty either towards the mouth or to the exterior (Fig. 2a, i).

**Fig. 3:**
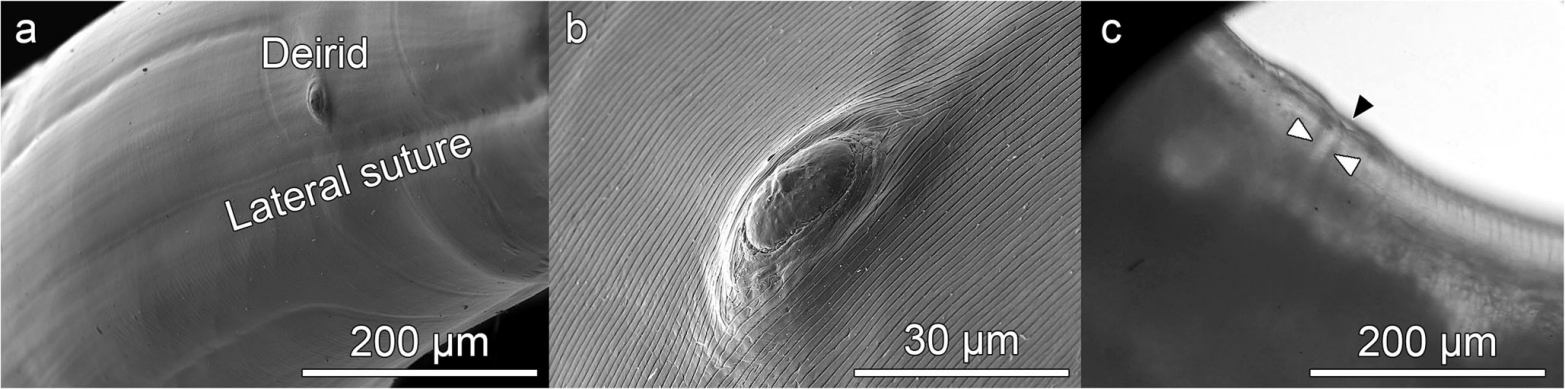
*Anisakis physeteris* L4. **a** Location of deirid and lateral suture near anterior end of larva. **b** Deirid. **c** Vagina (white arrowheads) and vulva (black arrowhead) by optical microscopy, located in anterior third of body

Near the anterior end of the L4, there are a pair of deirids, positioned symmetrically and subdorsally next to the lateral suture of the cuticle (Fig. 3a, b). These project from the cuticle and are oval and flat with a central button. They are surrounded by structured cuticle which emphasizes them. Their maximum diameter appears to increase slightly as the L4 develops, from around 16 mμ (1 week after molting) to around 27 mμ (9 weeks after molting). No centrids were observed in either of the larval stages. The vulva was observed by optical microscopy (Fig. 3c) in the first third of the body of some of the L4 females which had spent most time in culture. However, it was not possible to examine it by SEM, probably due to it being hidden beneath the cuticle.

### Posterior or caudal end

**Fig. 4:**
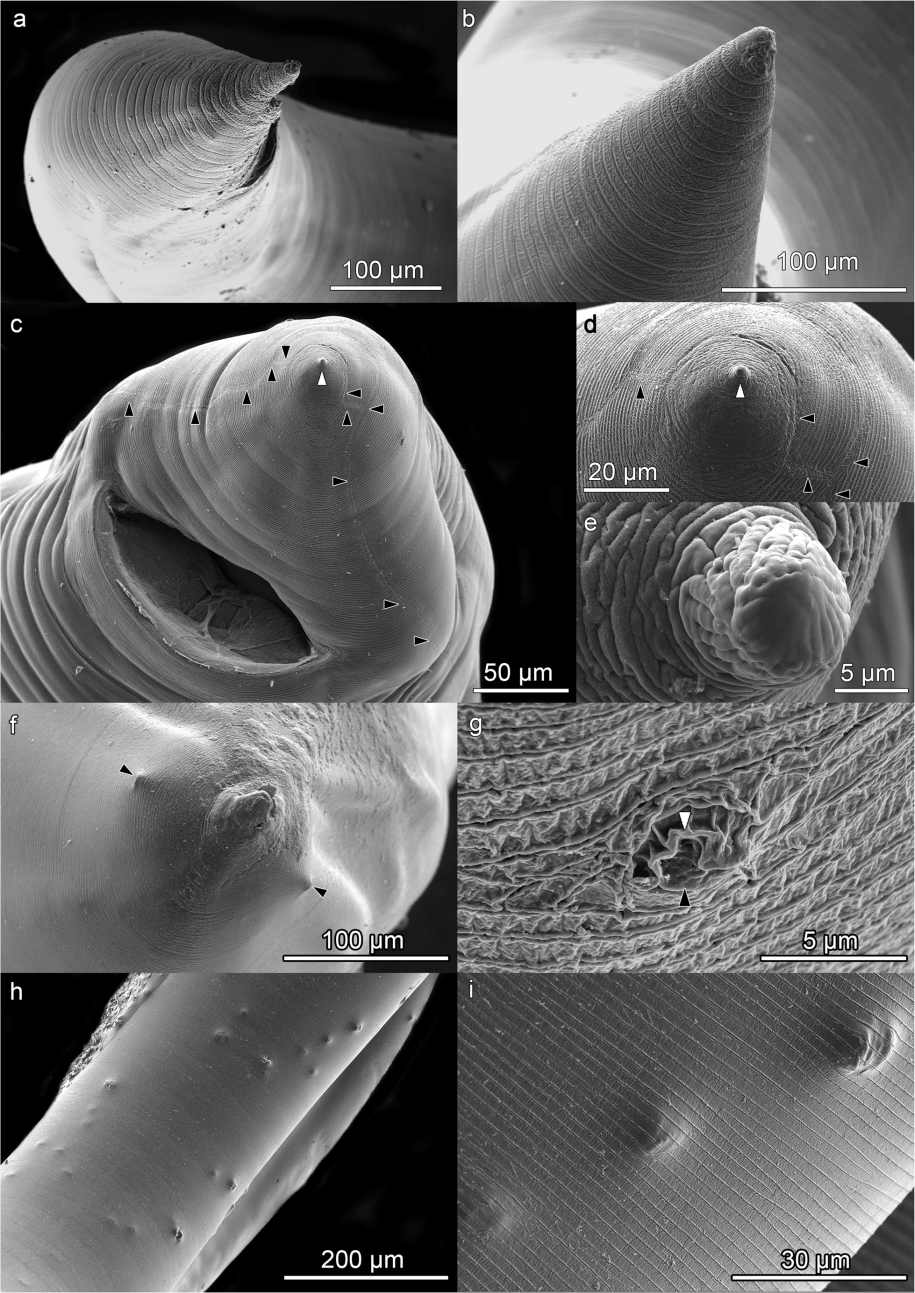
*Anisakis physeteris* larvae caudal end. **a** Anus and tip of L3. **b** Detail of end of tail of L3, note appearance of cuticle. **c** Anus and conical end of tail with blunt point (white arrowhead) of L4, note lateral suture (black arrowheads). **d** Conical end of tail of L4 with blunt point (white arrowhead), note lateral suture (black arrowheads). **e** Detail of blunt point at end of tail of L4, note appearance of cuticle. **f** View from above of end of tail of L4 showing the two phasmids (black arrowheads), lateral and symmetric. **g** Detail of a phasmid of L4, note the papilla (black arrowhead) surrounded by cuticle (white arrowhead). **h** Papilla-like structures in the ventral preanal zone of L4, beneath the cuticle as seen in **i**

The posterior end of L3 and L4 is similar (Fig. 4). In the former, it is conical with a rounded point with a “C”-shaped anus with the ends pointing towards the posterior end (Fig. 4a, b). No other structures were observed in L3. L4 showed an anus and tail similar to L3 although terminating in a conical lobe with a blunt point (Fig. 4c, d, e). The plectanes described in adults of *A. physeteris* (Davey 1971) were not observed.

Phasmids are other sensory organs located at the caudal end of adult nematodes of the class Secernentea. These have not yet been detected in L3 of *Anisakis* although we do not rule out that they could be formed beneath the cuticle, like the other sensory structures mentioned above. But one pair of phasmids were observed here throughout the L4 stage (Fig. 4f, g), as in other anisakids (Fagerholm 1991). These are symmetrical and located laterally at each side of the tail, unlike in other members of clade 3, such as *A. paggiae* where they are subventral (Di Azevedo et al. 2015) and *A. brevispiculata* where they have been reported as sublateral (Abollo and Pascual 2002). They are found closer to the point of the tail than to the anus and are separated from the surrounding cuticle by a groove (Fig. 4g). In some cases, a depression can be seen (Fig. 4f) but this may be due to the technique of sample preparation for SEM. Their diameter, around 5 mμ, does not seem to vary significantly during the development of L4. In some of the most well-developed L4, papilla-like bumps, beneath cuticle, could be observed in the preanal ventral zone. These may be the preanal papillae of the adult male (Fig. 4h, i).

### Cuticle

**Fig. 5:**
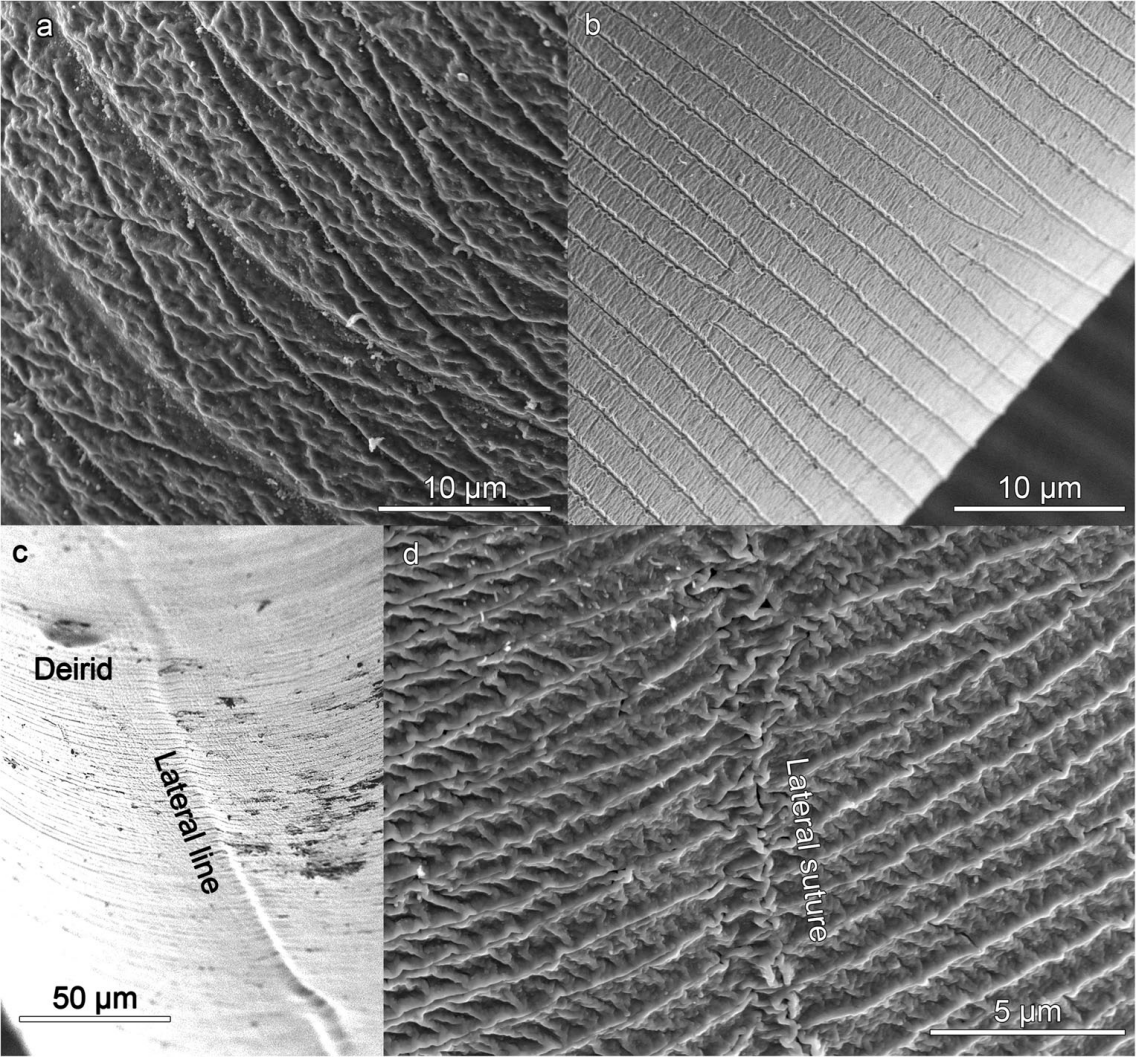
*Anisakis physeteris* cuticle. **a** Cuticle structure of posterior end of L3. **b** Cuticle structure of L4 in ventral preanal zone. **c** Detail of lateral line, in anterior zone of L3. **d** Detail of lateral suture, in posterior zone of L4

At the anterior end of the larva, the cuticle has an irregular structure. In the L3, it is smooth (Fig. 1) with some slight, irregularly distributed striations (Valter et al. 1982). In the L4, the surface is rougher, although appearing structured (Fig. 2). The internal face of the upper zone of the lips is smooth while the external face is rough (Fig. 2g). On the body of the L3 and L4 larvae, below the lips, the cuticle is striated and a cephalic collar is absent (Figs. 1a, f, and 2a). The striations are across the body and parallel to each other, distributed more or less regularly throughout the length of the worm (Figs. 1g, and 5). Occasionally, the cuticular annuli are subdivided (Fig. 5b), probably as part of the growth process of the cuticle (Molina-Fernández et al. 2017). Vertical bands can also be observed in them (Figs. 4b, and 5b). These annuli are similar to those described for type I L4 of *Anisakis* but are different to the irregular striations shown in type I L3 (Shiraki 1974; Weerasooriya et al. 1986). While in type I *Anisakis*, the width of the cuticular annuli increases from L3 to L4, in *Pseudoterranova decipiens*, it decreases (Shiraki 1974; Weerasooriya et al. 1986). In type II *Anisakis*, the width of the annuli at the anterior end of L3 is similar to that of L4. However, while the width of the annuli remains the same throughout the body of L4, in L3, the annuli at the posterior end are wider (7.3–9.0 mμ), similar to the case described by Tongu et al. (1990) (6.4–9.3 mμ). In L4, up to and including the third week, the annuli measure 1.0–1.1 mμ (tail zone) but later increase in width up to 1.4–1.9 mμ (> 3 weeks), although, in the zone closest to the conical lobe, they decrease to ~ 0.7 mμ. Although no lateral alae can be observed, a lateral line in the cuticle, without structural changes, can be observed in L3 (Fig. 5c) and a lateral suture in L4 (Fig. 5d) separating the dorsal portion from the ventral portion throughout the length of the nematode (Figs. 1f, 3a, and 5), except at the tail (Fig. 4a), where they are not visible in L3, although in L4 they reach the base of the conical lobe (Fig. 4c, d). The annuli at the end of the tail of the L3 exhibit a distinctive pattern (Fig. 5a), while, in the L4, the lobe and the blunt point have an irregular cuticle (Fig. 4d) which is clearly different from that of type I *Anisakis* which shows “a large number of spherical elevations” (Weerasooriya et al. 1986). The usefulness of cuticle structure for the identification of anisakid species and their developmental stages has been widely debated. Thus, while some authors such as van Thiel (1966) or Davey (1971) rejected its use, others such as Shiraki (1974) or Weerasooriya et al. (1986) considered that it could be useful, particularly for deteriorated specimens collected from a human patient where the morphological identification would be difficult. This problem has since been resolved by the development of molecular techniques (Zhu et al. 1998).

It is noteworthy that the sensory structures of one larval stage develop during the previous stage, remaining beneath the cuticle until molting, when they become visible. As they are sensory organs, they may not become functional until exposed to the external medium following molting, although, as mentioned previously, Jones (1994) suggested that they are already functional. This is certainly the case of cephalic papillae, amphids, and deirids of the L4, formed beneath the cuticle of the L3 or of the caudal papillae of the males, formed beneath the L4 cuticle. Further study will be required in order to answer these questions.
